# A Vision-Based Social Distancing and Critical Density Detection System for COVID-19

**DOI:** 10.3390/s21134608

**Published:** 2021-07-05

**Authors:** Dongfang Yang, Ekim Yurtsever, Vishnu Renganathan, Keith A. Redmill, Ümit Özgüner

**Affiliations:** Department of Electrical and Computer Engineering, The Ohio State University, Columbus, OH 43210, USA; yurtsever.2@osu.edu (E.Y.); renganathan.5@osu.edu (V.R.); redmill.1@osu.edu (K.A.R.); ozguner.1@osu.edu (Ü.Ö.)

**Keywords:** convolutional neural network, social distancing, pedestrian detection, linear regression

## Abstract

Social distancing (SD) is an effective measure to prevent the spread of the infectious Coronavirus Disease 2019 (COVID-19). However, a lack of spatial awareness may cause unintentional violations of this new measure. Against this backdrop, we propose an active surveillance system to slow the spread of COVID-19 by warning individuals in a region-of-interest. Our contribution is twofold. First, we introduce a vision-based real-time system that can detect SD violations and send non-intrusive audio-visual cues using state-of-the-art deep-learning models. Second, we define a novel critical social density value and show that the chance of SD violation occurrence can be held near zero if the pedestrian density is kept under this value. The proposed system is also ethically fair: it does not record data nor target individuals, and no human supervisor is present during the operation. The proposed system was evaluated across real-world datasets.

## 1. Introduction

With the outbreak of the novel Coronavirus Disease 2019 (COVID-19) [1], social distancing (SD) emerged as an effective measure against it. Maintaining social distancing in public areas such as transit stations, shopping malls, and university campuses is crucial to prevent or slow the spread of the virus. The practice of social distancing (SD) may continue in the following years until the spread of the virus is completely phased out. However, social distancing is prone to be violated unwillingly, as populations are not accustomed to keeping the necessary 2-meter bubble around each individual. This work proposes a vision-based automatic warning system that can detect social distancing statuses and identify a critical pedestrian density threshold to modulate inflow to crowded areas. Besides being an automated monitoring and warning system, the proposed framework can serve as a tool to detect key variables and statistics for local and global virus control.

Vision-based automatic detection and control systems [2,3,4,5,6,7,8,9] are economic and effective solutions to mitigate the spread of COVID-19 in public areas. Although the conceptualization is straightforward, the design and deployment of such systems require smart system design and serious ethical considerations.

First, the system must be fast and real-time. Only a real-time system can detect social distancing statuses immediately and send a warning. Privacy concerns [10,11,12] can be mitigated with a real-time system by not storing sensitive image data while only keeping aggregate statistics, such as the number of SD violations. With a real-time active surveillance system, appropriate measures can be taken as quickly as possible to reduce further spread of COVID-19.

The second design objective is that the system must be accurate and effective enough, but not discriminative. The safest way to achieve this is to build an AI-based detection system. AI-based vision detectors have become the state-of-the-art in people-detection tasks, achieving higher scores in most vision benchmarks than detectors with hand-crafted feature extractors. Furthermore, the latter may lead to maligned designs, whereas an end-to-end AI-based system, such as a deep neural network without any feature-based input space, is much fairer, with one caveat: the training data distribution must be fair.

The third objective aims to provide a more advanced measure than pure social distancing monitoring to further reduce the spread of COVID-19. This leads to our proposed approach of critical pedestrian density identification. The critical density may serve as an indicator to inform the space manager to control the entry port to regulate the incoming pedestrian flow. An online warning is also possible, but it should be non-alarming. For example, the system can send a non-intrusive audio-visual cue to the vicinity of the social distancing violation. Individuals in this region can then make their own decisions with this cue.

We identify the fourth design objective as trust establishment. The whole system and its implementation must be open-sourced. Open-sourcing is crucial for establishing trust between the active surveillance system and society. In addition, researchers and developers can freely and quickly access relevant material and further improve their own designs according to their requirements. This can hasten the development and deployment of anti-COVID-19 technologies, leading to stopping of the spread of the deadly disease and saving lives.

**Figure 1:**
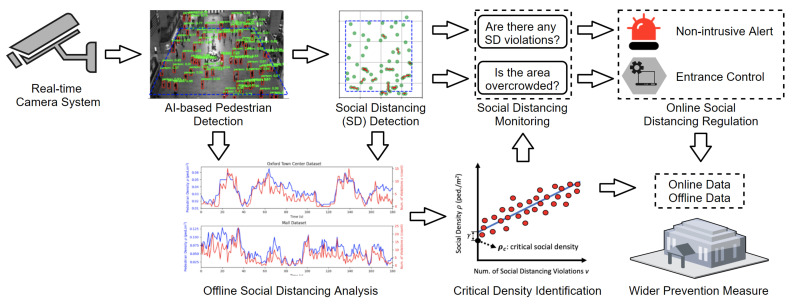
Overview of the proposed system. An audio-visual cue is emitted each time an individual breach of social distancing is detected. We also make a novel contribution by defining a critical social density value $\rho_{c}$ for measuring overcrowding. Entrance into the region-of-interest can be modulated online with this value. The aggregated non-personal data can also be analyzed offline to provide more insights into the social distancing practice in different public areas. Based on both online and offline data, wider prevention measures can be taken as quickly as possible when necessary. Our system is real-time and does not record data.

Against this backdrop, we propose a non-intrusive, AI-based active surveillance system for social distancing detection, monitoring, analysis, and control. The overview of the system is shown in Figure 1. The proposed system first uses a pre-trained deep convolutional neural network (CNN) [13,14] to detect individuals with bounding boxes in a given monocular camera frame. Then, detections in the image domain are transformed into real-world bird’s-eye-view coordinates for social distancing detection. Once the social distancing is detected, information is passed to two branches for further processing. One branch is online monitoring and control. If a social distancing violation happens, the system emits a non-alarming audio-visual cue. Simultaneously, the system measures social (pedestrian) density. If the social density is larger than a critical threshold, the system sends an advisory inflow modulation signal to prevent overcrowding. The other branch is offline analysis, which provides necessary information for overcrowding prevention and policymaking. The main analysis is the identification of a critical social density. If the pedestrian density is regulated under this critical value, the probability of social distancing violations will be kept near zero. Finally, the regulator can receive both the offline aggregate statistics and the online status of social distancing control. If immediate action is required, the regulator can act as quickly as possible.

The overall system never stores personal information. Only the processed average results, such as the number of violations and pedestrian density, are stored. This is extremely important for privacy concerns. Our system is also open-sourced for further development.

Our main contributions are:

- A novel, vision-based, real-time social distancing and critical social density detection system.
- Definition of critical social density and a statistical approach to measuring it.
- Measurements of social distancing and critical density statistics of common crowded places, such as the New York Central Station, an indoor mall, and a busy town center in Oxford.
- Quantitative validation of the proposed approach to detect social distancing and critical density.

## 2. Related Work

**Social distancing for COVID-19.** COVID-19 has caused severe acute respiratory syndromes around the world since December 2019 [15]. Social distancing is an effective measure to slow the spread of COVID-19 [1], which is defined as keeping a minimum of 2 meters (6 feet) apart from other individuals to avoid possible contact. Further analysis [16] also suggests that social distancing has substantial economic benefits. COVID-19 may not be completely eliminated in the short term, but an automated system that can help in the monitoring and analyzing social distancing measures can greatly benefit our society. Statistics from recent works [1] have demonstrated that strong social distancing measures can indeed reduce the growth rate of COVID-19.

The requirement of social distancing has shaped the development of IoT sensors and smart city technologies. The spread prevention and outbreak alerting of COVID-19 now must be considered in these areas. A recent work [17] reviews potential solutions and recent approaches, such as IoT sensors, social media, personal gadgets, and public agents for COVID-19 outbreak alerting. Another work [18] summarises IoT and associated sensor technologies for virus tracing, tracking, and spread mitigation, and highlights the challenges of deploying such sensor hardware.

With the help of the above technologies, social distancing can be better practiced, which will eventually alleviate the spread of the virus and “flatten the curve”.

**Social distancing monitoring.** In public areas, social distancing is mostly monitored by vision-based IoT systems with pedestrian detection capabilities. Appropriate measures are subsequently taken on this basis.

Pedestrian detection can be viewed as a sub-task of generic object detection or as a specific task of detecting pedestrians only. A detailed survey of 2D object detectors and the corresponding datasets, metrics, and fundamentals can be found in [19]. Another survey [20] focuses on deep-learning-based approaches for both the generic object detectors and the pedestrian detectors. Generally speaking, state-of-the-art detectors are divided into two categories. One category is two-stage detectors. Most of them are based on R-CNN [13,21], which starts with region proposals and then performs the classification and bounding box regression. The other category is one-stage detectors. Prominent models include YOLO [14,22,23], SSD [24], and EfficientDet [25]. The detectors can also be classified as anchor-based [13,14,21,23,24,25] or anchor-free approaches [26,27]. The major difference between them is whether to use a set of predefined bounding boxes as candidate positions for the objects. Evaluating these approaches was usually done using the datasets of Pascal VOC [28] and MS COCO [29]. The accuracy and real-time performance of these approaches are good enough for deploying pre-trained models for social distancing detection.

There are several emerging technologies that assist in the practice of social distancing. A recent work [2] has identified how emerging technologies like wireless, networking, and artificial intelligence (AI) can enable or even enforce social distancing. The work discussed possible basic concepts, measurements, models, and practical scenarios for social distancing. Another work [3] has classified various emerging techniques as either human-centric or smart-space categories, along with the SWOT analysis of the discussed techniques. Social distancing monitoring is also defined as a visual social distancing (VSD) problem in [4]. The work introduced a skeleton-detection-based approach for inter-personal distance measuring. It also discussed the effect of social context on people’s social distancing and raised the concern of privacy. The discussions are inspirational, but again, do not generate solid results for social distancing monitoring and leaves the question open. A specific social distancing monitoring approach [5] that utilizes YOLOv3 and Deepsort was proposed to detect and track pedestrians followed by calculating a violation index for non-social-distancing behaviors. The approach is interesting, but the results do not contain any statistical analysis. Furthermore, there is no implementation or privacy-related discussion other than the violation index. Another work [6] developed a DNN model called DeepSOCIAL for people detection, tracking, and distance estimation. In addition to social distancing monitoring, it also performed dynamic risk assessment. However, this work did not specifically consider the performance of pure violation detection and the solution to prevent overcrowding. More recently, [30] provides a data-driven deep-learning-based framework for the sustainable development of a smart city. Some other works [7,8,9] also proposed vision-based solutions. A comparison of the above methods with our proposed method can be found in Table 1.

Several prototypes utilizing machine learning and sensing technologies have already been developed. Landing AI [31] was almost the first one to introduce a social distancing detector using a surveillance camera to highlight people whose physical distance is below the recommended value. A similar system [9] was deployed to monitor worker activity and send real-time voice alerts in a manufacturing plant. In addition to surveillance cameras, LiDAR-based [32] and stereo-camera-based [33] systems were also proposed, which demonstrated that different types of sensors besides surveillance cameras can also help.

The above systems are interesting, but recording data and sending intrusive alerts might be unacceptable by some people. On the contrary, we propose a non-intrusive warning system with softer omnidirectional audio-visual cues. In addition, our system evaluates critical social density and modulates inflow into a region-of-interest.

## 3. Preliminaries

**Object detection with deep learning.** Object detection in the image domain is a fundamental computer vision problem. The goal is to detect instances of semantic objects that belong to certain classes, such as humans, cars, and buildings. Recently, object detection benchmarks have been dominated by deep Convolutional Neural Network (CNN) models [13,14,21,23,24,25]. For example, top scores on MS COCO [29], which has over 123K images and 896K objects in the training-validation set and 80K images in the testing set with 80 categories, have almost doubled thanks to the recent breakthrough in deep CNNs.

These models are usually trained by supervised learning, with techniques like data augmentation [34] to increase the variety of data.

**Model generalization.** The generalization capability [35] of the state-of-the-art is good enough for deploying pre-trained models to new environments. For 2D object detection, even with different camera models, angles, and illumination conditions, pre-trained models can still achieve good performance.

Therefore, a pre-trained state-of-the-art deep-learning-based pedestrian detector can be directly utilized for the task of social distancing monitoring.

## 4. Method

We propose to use a fixed monocular camera to detect individuals in a region of interest (ROI) and measure the inter-personal distances in real time without data recording. The proposed system sends a non-intrusive audio-visual cue to warn the crowd if any social distancing breach is detected. Furthermore, we define a novel critical social density metric and propose to advise not entering into the ROI if the density is higher than this value. The overview of our approach is given in Figure 1, and the formal description starts below.

### 4.1. Problem Formulation

We define a scene at time *t* as a sextuple $S=(I,A_{0},d_{c},c_{1},c_{2},U_{0})$, where $I\in R^{H\times W\times 3}$ is an RGB image captured from a fixed monocular camera with height *H* and width *W*. $A_{0}\in R$ is the area of the ROI on the ground plane in the real world and $d_{c}\in R$ is the required minimum physical distance. $c_{1}$ is a binary control signal for sending a non-intrusive audio-visual cue if any inter-pedestrian distance is less than $d_{c}$. $c_{2}$ is another binary control signal for controlling the entrance to the ROI to prevent overcrowding. Overcrowding is detected with our novel definition of critical social density $\rho_{c}$. $\rho_{c}$ ensures the social distancing violation occurrence probability stays lower than $U_{0}$. The threshold $U_{0}$ should be set as small as possible to reduce the probability of social distancing violation. For example, the threshold could be $U_{0}=\frac{1-P_{\mathrm{CI}}}{2}$, where $P_{\mathrm{CI}}$ is the cumulative probability of the 95% confidence interval of a normal distribution. Other choices of $U_{0}$ also work, depending on the specific requirement of social distancing monitoring.

**Problem** **1.** *Given S, we are interested in finding a list of pedestrian position vectors $P=(p_{1},p_{2},\cdots ,p_{n})$, $p\in R^{2}$, in real-world coordinates on the ground plane and a corresponding list of inter-pedestrian distances $D=(d_{1,2},\cdots ,d_{1,n},d_{2,3},\cdots ,d_{2,n},\cdots ,d_{n-1,n})$, $d\in R^{+}$. n is the number of pedestrians in the ROI. Additionally, we are interested in finding a critical social density value $\rho_{c}$. $\rho_{c}$ should ensure the probability $p(d>d_{c}|\rho <\rho_{c})$ stays over $1-U_{0}$, where we define social density as $\rho :=n/A_{0}$.*

Once Problem 1 is solved, the following control algorithm can be used to warn/advise the population in the ROI.

**Algorithm** **1.** If $d\leq d_{c}$, then a non-intrusive audio-visual cue is activated with setting the control signal $c_{1}=1$, otherwise $c_{1}=0$. In addition, if $\rho >\rho_{c},$ then entering the area is not advised with setting $c_{2}=1$, otherwise $c_{2}=0$.

Our solution to Problem 1 starts below.

### 4.2. Pedestrian Detection in the Image Domain

First, pedestrians are detected in the image domain with a deep CNN model trained on a real-world dataset:

(1) $${\left\{T_{i}\right\}}_{k}=f_{\mathrm{cnn}}\left(I\right).$$

$f_{\mathrm{cnn}}:I\to {\left\{T_{i}\right\}}_{n}$ maps an image I into *n* tuples $T_{i}=\left(l_{i}b_{i},s_{i}\right),\forall i\in \left\{1,2,\cdots ,n\right\}$. *n* is the number of detected objects. $l_{i}\in L$ is the object class label, where *L*, the set of object labels, is defined in $f_{\mathrm{cnn}}$. $b_{i}=\left(b_{i,1},b_{i,2},b_{i,3},b_{i,4}\right)$ is the associated bounding box (BB) with four corners. $b_{i,j}=\left(x_{i,j},y_{i,j}\right)$ gives pixel indices in the image domain. The second sub-index *j* indicates the corners at top-left, top-right, bottom-left, and bottom-right, respectively. $s_{i}$ is the corresponding detection score. Implementation details of $f_{\mathrm{cnn}}$ is given in Section 5.1.

We are only interested in the case of l= ‘person’. We define $p_{i}^{\prime }$, the pixel pose vector of person *i*, by using the middle point of the bottom edge of the BB:

(2) $$p_{i}^{\prime }:=\frac{\left(b_{i,3}+b_{i,4}\right)}{2}.$$

### 4.3. Image to Real-World Mapping

The next step is obtaining the second mapping function $h:p^{\prime }\to p$. *h* is an inverse perspective transformation function that maps $p^{\prime }$ in image coordinates to $p\in R^{2}$ in real-world coordinates. p is in 2D bird’s-eye-view (BEV) coordinates by assuming the ground plane z=0. We use the following well-known inverse homography transformation [36] for this task:

(3) $$p^{\mathrm{bev}}=M^{-1}p^{\mathrm{im}},$$

where $M\in R^{3\times 3}$ is a transformation matrix describing the rotation and translation from world coordinates to image coordinates. $p^{\mathrm{im}}=\left[p_{x}^{\prime },p_{y}^{\prime },1\right]$ is the homogeneous representation of $p^{\prime }=\left[p_{x}^{\prime },p_{y}^{\prime }\right]$ in image coordinates, and $p^{\mathrm{bev}}=\left[p_{x}^{\mathrm{bev}},p_{y}^{\mathrm{bev}},1\right]$ is the homogeneous representation of the mapped pose vector.

The transformation matrix M can be found by identifying the geometric relationship among some key points in both the real world and the image, respectively, and then calculating M based on homography [36]. More details on camera calibration in this particular work can be found in Section 5.1.

The world pose vector p is derived from $p^{\mathrm{bev}}$ with $p=[p_{x}^{\mathrm{bev}},p_{y}^{\mathrm{bev}}]$.

### 4.4. Social Distancing Detection

After getting $P=(p_{1},p_{2},\cdots ,p_{n})$ in real-world coordinates, obtaining the corresponding list of inter-pedestrian distances *D* is straightforward. The distance $d_{i,j}$ for pedestrians *i* and *j* is obtained by taking the Euclidean distance between their pose vectors:

(4) $$d_{i,j}=∥p_{i}-p_{j}∥.$$

The total number of social distancing violations *v* in a scene can be calculated by:

(5) $$v=\sum_{i=1}^{n}\sum_{\begin{array}{c} j=1\\ j\neq i \end{array}}^{n}I\left(d_{i,j}\right),$$

where $I(d_{i,j})=1$ if $d_{i,j}<d_{c}$, otherwise 0.

### 4.5. Critical Social Density Estimation

Finally, we want to find a critical social density value $\rho_{c}$ that can ensure the social distancing violation occurrence probability stays below $U_{0}$. It should be noted that a trivial solution of $\rho_{c}=0$ will ensure v=0, but it has no practical use. Instead, we want to find the maximum critical social density $\rho_{c}$ that can still be considered safe.

To find $\rho_{c}$, we propose to conduct a simple linear regression using the social density ρ as the dependent variable and the total number of violations *v* as the independent variable:

(6) $$\rho =\beta_{0}+\beta_{1}v+\epsilon ,$$

where $\beta =[\beta_{0},\beta_{1}]$ is the regression parameter vector and ϵ is the error term which is assumed to be normal. The regression model is fitted with the ordinary least squares method. Fitting this model requires training data. However, once the model is learned, data are not required anymore. After deployment, the surveillance system operates without recording data.

**Figure 2:**
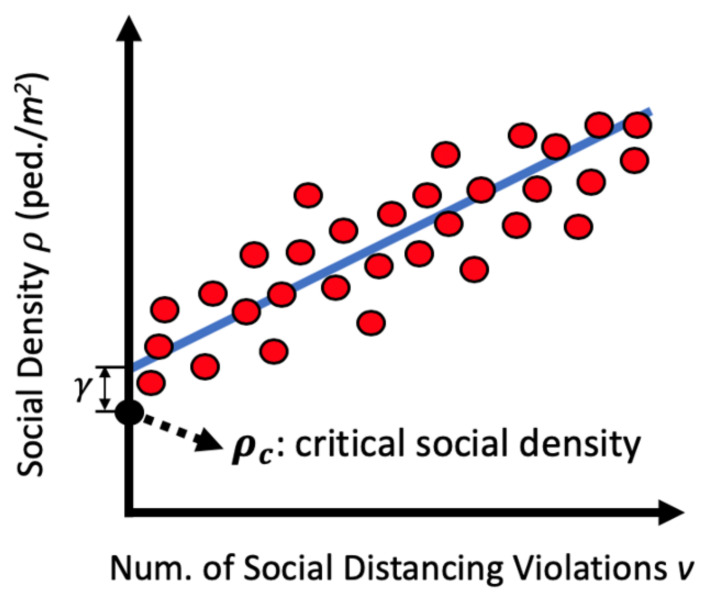
Obtaining the critical social density $\rho_{c}$. Keeping ρ under $\rho_{c}$ will drive the number of social distancing violations *v* towards zero with the linear regression assumption.

Once the model is fitted, we can obtain the predicted social density $\hat{\rho }{|}_{v=0}$ when there is no social distancing violation (v=0). To further reduce the probability of social distancing violation occurrence, instead of using $\hat{\rho }{|}_{v=0}$, we propose to determine the critical social density as:

(7) $$\rho_{c}=\rho_{\mathrm{lb}}^{\mathrm{pred}},$$

where $\rho_{\mathrm{lb}}^{\mathrm{pred}}$ is the lower bound of the 95% prediction interval $\left(\rho_{\mathrm{lb}}^{\mathrm{pred}},\rho_{\mathrm{ub}}^{\mathrm{pred}}\right)$ at v=0, as illustrated in Figure 2.

If we keep the social density ρ of a scene to be smaller than the lower bound $\rho_{\mathrm{lb}}$ of the social density’s 95% prediction interval at v=0, the probability of social distancing violation occurrence can be pushed near zero. This is because under the linear regression assumption, the cumulative probability $P(\rho <\rho_{\mathrm{lb}}^{\mathrm{pred}})=0.05$, which is very small.

### 4.6. Broader Implementation

To further utilize the obtained critical social density $\rho_{c}$, subsequent measures must be taken to prevent the spread of COVID-19. There are two branches of post-processing mechanisms.

First, social distancing can be monitored and controlled online. Non-alarming audio-visual cues are sent to the people in the areas where the social density ρ is larger than the critical value $\rho_{c}$. In this way, people are immediately aware that they are violating the social distancing practice. The system can also send inflow modulation signals. Site managers can use these signals to keep the people density under $\rho_{c}$. This way, overcrowding is prevented.

Second, the critical density $\rho_{c}$, as well as the statistics, can be used for offline analysis. Analyzed offline data, such as averaged people densities of certain public areas or trends of people density of public events, can be utilized by regulators for better policymaking and large event organization.

Combining both the offline and online information provided by the proposed system, wider prevention measures can be taken as quickly as possible when necessary. The above procedures can be visualized in Figure 1.

## 5. Experiments

We conducted three case studies to evaluate the proposed method. Each case utilizes a different pedestrian crowd dataset. They are the Oxford Town Center Dataset (an urban street) [37], the Mall Dataset (an indoor mall) [38], and the Train Station Dataset (New York City Grand Central Terminal) [39]. Table 2 shows detailed information about these datasets.

To validate the effectiveness of the proposed method in detecting social distancing violation, we conducted experiments over Oxford Town Center Dataset to determine the accuracy of the proposed method.

### Implementation

The first step was finding the perspective transformation matrix M for the scene of each dataset. For the Oxford Town Center Dataset, we directly used the transformation matrix available on its official website. The other two datasets do not provide the transformation matrices, so we need to find them manually. We first identified the real distances among four key points in the scene and the corresponding coordinates of these points in the image. Then, these four points were used to identify the perspective transformation (homography) [36] so that the transformation matrix M can be calculated. For the Train Station Dataset, we found the floor plan of NYC Grand Central Terminal and measured the exact distances among the key points. For the Mall Dataset, we first estimated the size of a reference object in the image by comparing it with the width of detected pedestrians and then utilized the key points of the reference object.

The second step was applying the pedestrian detector on each dataset. The experiments were conducted on a regular PC with an Intel Core i7-4790 CPU, 32GB RAM, and an Nvidia GeForce GTX 1070Ti GPU running Ubuntu 16.04 LTS 64-bit operating system. Once the pedestrians were detected, their positions were converted from the image coordinates into the real-world coordinates.

The last step was conducting the social distancing measurement and finding the critical density $\rho_{c}$. Only the pedestrians within the ROI were considered. The statistics of the social density ρ, the inter-pedestrian distances $d_{i,j}$, and the number of violations *v* were recorded over time.

## 6. Results

### 6.1. Real-Time Pedestrian Detection

**Figure 3:**
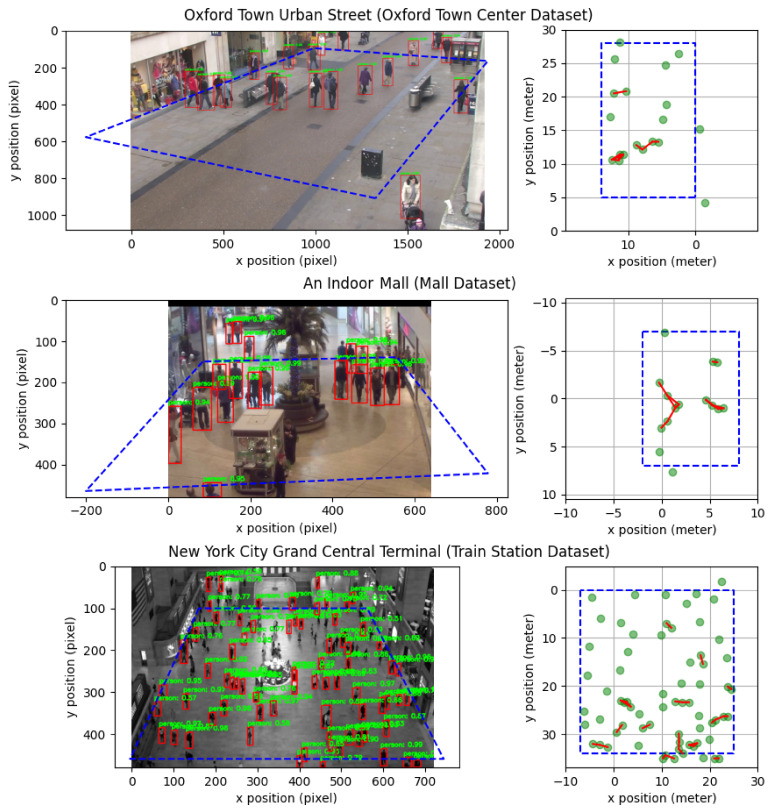
Illustration of pedestrian detection using Faster R-CNN [13] and the corresponding social distancing.

We experimented with two different deep-CNN-based object detectors: Faster R-CNN and YOLOv4. Figure 3 shows the qualitative results of pedestrian detection in the image using Faster R-CNN [13] and the corresponding social distancing in world coordinates. According to the qualitative results, there are a few missed detections. The reasons could be two-fold. First, occlusions can cause missed detections. This can be found in Mall Dataset, in which the shopping cart may affect the detection. Second, if the pedestrian size is too small, missed detections may also happen. This can be found in the Train Station Dataset. A limited number of missed detections do not affect the social distancing violation too much, as the first priority of the system is to detect whether there is any social distancing violation. For the number of violations and critical social density, as long as we can find a close enough estimation, it will satisfy our requirement.

The detector performances are given in Table 3. As can be seen in the Table, both detectors achieved an inference time of about 0.1s per frame. This is adequate to achieve real-time social distancing detection. For detection accuracy, we provide the results of MS COCO dataset from original works [13,14].

### 6.2. Social Distancing Violation Detection

**Figure 4:**
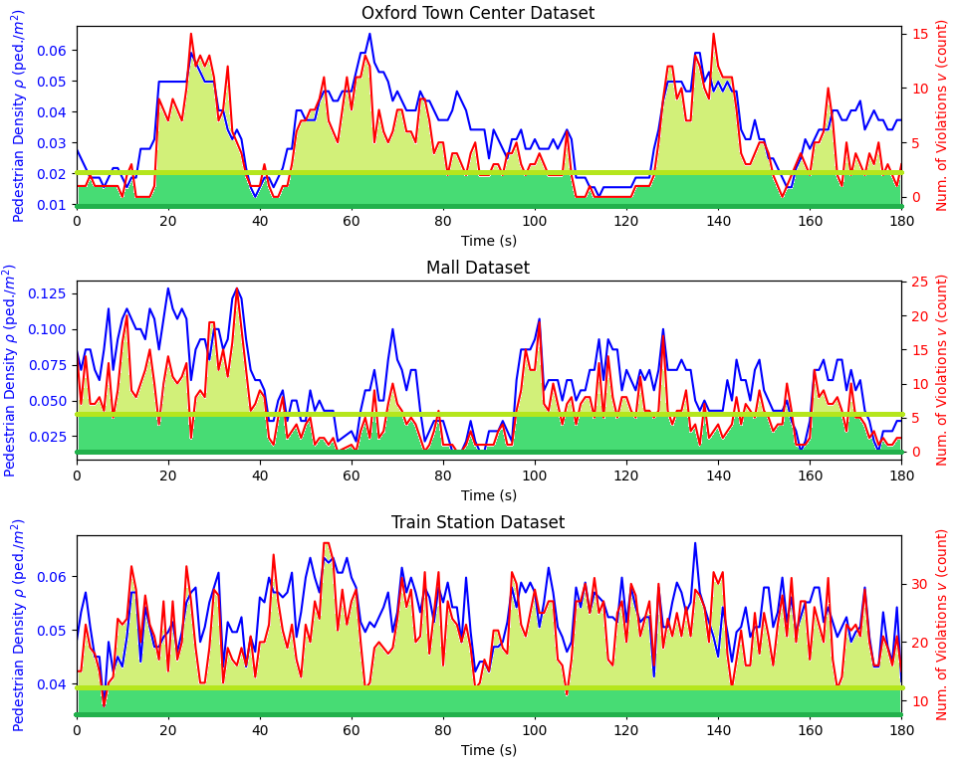
The change of pedestrian density ρ and the number of violations *v* over time. It shows an obvious positive correlation between ρ and *v*. The positive correlation is further illustrated in Figure 5 and Figure 6, which show a linear relationship between ρ and *v*. The darker green horizontal line indicates the critical pedestrian density $\rho_{c}$ and the lighter green line, the intercept density $\beta_{0}$. They are obtained by the proposed critical social density estimation methodology in Section 4.5. The shaded lighter green area shows that there will be more violations if the pedestrian density is above $\beta_{0}$. The shaded darker green area shows that more violations will be eliminated if the pedestrian density is further pushed below $\rho_{c}$, which is our critical social density.

**Figure 5:**
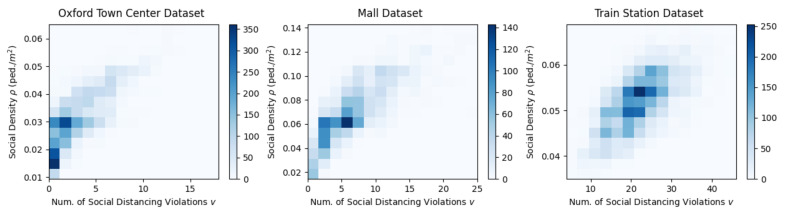
Two-dimensional histograms of the social density ρ versus the number of social distancing violations *v*. From the histograms we can see a linear relationship with positive correlation.

Figure 4 shows the change of pedestrian density ρ and the number of violations *v* as time evolves. The result indicates an obvious positive correlation between ρ and *v*. For example, at t=41 s in the Oxford Town Center Dataset, t=84 s in the Mall Dataset, and t=6 s in the Train Station Dataset, when ρ is low, *v* is also relatively low. Figure 5 shows 2D histograms of this relationship. It further validates the observed positive correlation. This correlation leads to the subsequent proposed linear regression method to identify critical social density.

To validate our proposed methodology, we conducted the evaluation over the Oxford Town Center dataset, as it provides ground truth pedestrian detection. There are, in total, 4501 annotated frames. We split them into two parts, 2500 frames for training and 2001 frames for validation. The reason for splitting the dataset is to compare the proposed method with an end-to-end CNN model for social distancing violation detection, which was trained based on the training frames. All the evaluation results used the validation frames.

We first calculated the mean absolute error (MAE) of the average closest physical distance $d_{\mathrm{avg}}$ over all frames. $d_{\mathrm{avg}}$ is calculated by $d_{\mathrm{avg}}=\frac{1}{n}\sum_{i=1}^{n}d_{i}^{\min}$, where $d_{i}^{\min}=\min\left(d_{i,j}\right)$, ∀j≠i∈{1,2,⋯n} is the closest physical distance for a particular pedestrian *i*. We also calculated the MAE of the social distancing violation ratio $r_{v}=v/n$, where *n* is the number of pedestrians inside the ROI. The results were compared with a variant of our method which uses the center of the detected BB as the pedestrian position (BB-center method) instead of the middle point of the bottom edge, as described in Section 4.2 (BB-bottom method).

Table 4 reports the MAE of $d_{\mathrm{avg}}$ and the MAE of $r_{v}$. It quantifies the error in the detection of physical distance and social distancing violations. The proposed BB-bottom method has relatively low MAEs and is better than its variant BB-center method.

Furthermore, the social distancing violation detection (the number of violations v>0) was evaluated in terms of the precision, recall, and accuracy against the ground truth violation. In addition to the comparison with the variant method using the BB center, we also tried an end-to-end CNN model. Specifically, the CNN model inputs the image frame and outputs whether there is any social distancing violation or not. This new model employs ResNet50 as a backbone and has additional layers for social distancing violation detection. The loss is defined as weighted binary cross-entropy. The model was trained based on the first 2500 frames. The model performance was tested based on the remaining 2001 frames, which provides a fair comparison with the other two methods.

Table 5 shows the confusion matrix of social distancing violation detection using the BB-bottom method. In the Oxford Town Center Dataset, social distancing violation happens in the majority of the frames, so the number of true positives dominates the confusion matrix. Table 6 reports precision, recall, and accuracy of the violation detection and compares them over the methods of End-to-End CNN, BB-center, and BB-bottom. The result shows that the BB-bottom method performs better than the other two methods in all three metrics. The other two methods are not able to balance between the precision and recall metrics. End-to-end CNN has relatively high recall, but the precision is not good enough. BB-center has relatively high precision, but low recall. This further demonstrates the effectiveness of using pre-trained pedestrian detectors in the image domain and transforming the middle point of the BB bottom edge as the pedestrian position into world coordinates.

### 6.3. Critical Social Density

To find the critical density $\rho_{c}$, we first investigated the relationship between the number of social distancing violations *v* and the social density ρ in 2D histograms, as shown in Figure 5. As can be seen in the Figure, *v* increases with an increase in ρ, which shows a linear relationship with a positive correlation. This indicates that the proposed linear regression can be used.

**Figure 6:**
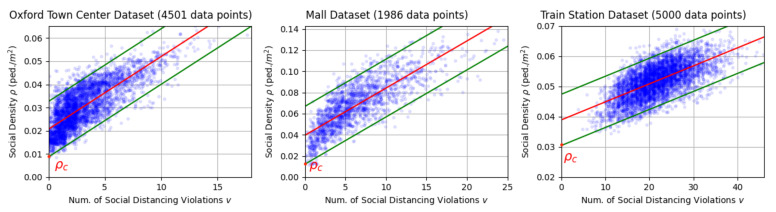
Linear regression (red line) of the social density ρ versus number of social distancing violations *v* data. Small random noise was added to each data point for better visualization. Green lines indicate the prediction intervals. The critical social densities $\rho_{c}$ are the x-intercepts of the regression lines. Data points might overlap.

Then, we conducted the linear regression using the regression model of Equation (6), on the data points of *v* versus ρ. The skewness values of ρ for the Oxford Town Center Dataset, Mall Dataset, and Train Station Dataset are 0.32, 0.16, and −0.14, respectively, indicating the distributions of ρ are symmetric. This satisfies the normality assumption of the error term in linear regression. The regression result is displayed in Figure 6. The critical density $\rho_{c}$ was identified as the lower bound of the prediction interval at v=0. As can be seen from the Figure, for a social density value ρ that is smaller than the lower bound $\rho_{c}$, there are almost no data points. This means that according to the scene in the dataset, when v=0, pedestrian density is hardly ever smaller than $\rho_{c}$. Since $\rho_{c}$ is the lower bound of the 95% prediction interval, if we keep a social density $\rho <\rho_{c}$, we can push the probability of social distancing violation to almost zero.

Table 7 summarises the identified critical densities $\rho_{c}$, as well as the intercepts $\beta_{0}$ of the regression models. The obtained critical density values for all datasets are similar. They also follow the patterns of the data points as illustrated in Figure 6. This verified the effectiveness of our method.

To evaluate the effect of social distancing detection on determining the critical density, we also conducted the linear regression on the data of ground truth pedestrian positions in the Oxford Town Center Dataset. The obtained regression result over ground truth pedestrian positions are $\beta_{0,\mathrm{gt}}=0.0217$ and $\rho_{c,\mathrm{gt}}=0.0086$. The critical density $\rho_{c}$ only has an error of 2%, which is very small. This further validated our proposed method of determining critical social density.

## 7. Conclusions

This work proposed an AI- and monocular-camera-based real-time system to detect and monitor social distancing. In addition, our system utilized the proposed critical social density value to avoid overcrowding by modulating inflow to the ROI. The proposed approach was demonstrated using three different pedestrian crowd datasets. Quantitative validation was conducted over the Oxford Town Center Dataset that provides ground truth pedestrian detections.

There were some missed detections in the Mall Dataset and Train Station Dataset, as in some areas the pedestrian density is extremely high and occlusions occur. However, after our qualitative and quantitative analysis, most pedestrians were successfully captured and the missed detections have an minor effect on the proposed method. One future activity could be testing and verifying the proposed method over more datasets of various scenes.

Finally, in this work we did not consider that a group of people might belong to a single family or have some other connection that does not require social distancing. Understanding and addressing this issue could be a futher direction of study. Nevertheless, one may argue that even individuals who have close relationships should still try to practice social distancing in public areas.

## Figures and Tables

**Table 1:** Comparison of vision-based social distancing detection.

Work	CC	AVS	RPDE	SDDE	OC
Khandelwal et. al. [9]	Yes		Yes		
DeepSOCIAL [6]	Yes	Yes	Yes		
Ahmed et. al. [7]			Yes		
Cota [8]	Yes		Yes	Yes	
Ours	Yes	Yes	Yes	Yes	Yes

Acronyms: camera calibration (CC), applicable to various scenes (AVS), real-time pedestrian detection evaluation (RPDE), social distancing detection evaluation (SDDE), overcrowding control (OC). This table compares the features that are relevant to this work. Some works may provide additional features.

**Table 2:** Information of each pedestrian dataset.

	FPS	Resolution	Duration
Oxford Town Ctr.	25	1920×1080	5 mins
Mall	∼1	640×480	33 mins
Train Station	25	720×480	33 mins

**Table 3:** Real-time performance of pedestrian detectors.

Method	mAP (%)	Inference Time (s)
Faster R-CNN [13]	42.1–42.7	0.145/0.116/0.108
YOLOv4 [14]	41.2–43.5	0.048/0.050/0.050

The mAP indicates mean average precision. The inference time reports the mean inference time for Oxford Town Center/Train Station/Mall datasets, respectively.

**Table 4:** Social distancing detection performance.

Method	MAE of $d_{\mathrm{avg}}$ (m)	MAE of $r_{v}$ (Count)
BB-center	1.416	0.196
BB-bottom	**0.587**	**0.143**

**Table 5:** Confusion matrix of social distancing violation detection.

		Ground Truth		Total
		Violation	No Violation	
Detected	Violation	1584	77	1661
	No Violation	67	273	340
	Total	1651	350	2001

The table reports the results of the BB-bottom method.

**Table 6:** Social distancing violation detection accuracy.

Method	Precision (%)	Recall (%)	Accuracy (%)
End-to-end CNN	83.27	94.37	79.71
BB-center	94.60	79.59	79.41
BB-bottom	**95.36**	**95.94**	**92.80**

**Table 7:** Critical social density detection.

Dataset	Intercept $\beta_{0}$	Critical Density $\rho_{c}$
Oxford Town Ctr.	0.0207	0.0088
Mall	0.0396	0.0123
Train Station	0.0389	0.0305

The critical density was identified as the lower bound of the prediction interval at the number of social distancing violations v=0.

## Data Availability

Our system is open-sourced. The implementation and the experiment data can be assessed via our GitHub repository: https://github.com/dongfang-steven-yang/social-distancing-monitoring, accessed on 2 July 2021.
